# Patterns of ongoing thought in the real world and their links to mental health and well-being

**DOI:** 10.1371/journal.pmen.0000309

**Published:** 2025-08-27

**Authors:** Bridget Mulholland, Louis Chitiz, Raven Wallace, Brontë Mckeown, Michael Milham, Arno Klein, Robert Leech, Elizabeth Jefferies, Giulia Poerio, Jeffrey Wammes, Jeremy Stewart, Samyogita Hardikar, Jonathan Smallwood

**Affiliations:** 1 Department of Psychology, Queen’s University, Kingston, Canada; 2 National Institute of Teaching, London, England; 3 Child Mind Institute, New York City, New York, United States of America; 4 Nathan S. Kline Institute for Psychiatric Research, Orangeburg, New York, United States of America; 5 Institute of Psychiatry, Psychology and Neuroscience, King’s College London, London, England; 6 Department of Psychology, University of York, York, England; 7 School of Psychology, University of Sussex, Falmer, England; Universidad del Valle, COLOMBIA

## Abstract

The thoughts we experience in daily life have implications for our mental health and well-being. However, it is often difficult to measure thought patterns outside of laboratory conditions due to concerns about the voracity of measurements taken in daily life. To address this gap in the literature, our study set out to measure patterns of thought as they occur in daily life and assess the robustness of these measures and their associations with trait measurements of mental health and well-being. A sample of undergraduate participants completed multi-dimensional experience sampling surveys eight times per day for five days as they went around their normal lives. Principal Component Analysis reduced these data to identify the dimensions that explained the patterns of thought reported by our participants. We used linear modelling to map how these thought patterns related to the activities taking place at the time of the probe, highlighting good consistency within the sample, as well as substantial overlap with prior work. Multiple regression was used to examine associations between patterns of ongoing thought and aspects of mental health and well-being, highlighting a pattern of ‘Intrusive Distraction’ that had a positive association with anxiety, and a negative association with social well-being. Notably, this thought pattern tended to be most prevalent in solo activities and was relatively suppressed when interacting with other people (either in person or virtually). Our study, therefore, highlights the use of multi-dimensional experience sampling as a tool to understand how ongoing thought in daily life impacts on our mental health and well-being and establishes the important role social connectedness plays in the etiology of intrusive thinking.

## Introduction

A fundamental goal of psychology is to understand how the thoughts we experience in daily life relate to our productivity, and mental health and well-being. Contemporary work indicates a link between patterns of ongoing thought, as determined by experience sampling, and various aspects of health and well-being (e.g., [1,2]). However, research on human thought has historically relied on observations made in controlled laboratory contexts, such as brain imaging (e.g., [3]) or behavioral laboratories (e.g., [4]), because of the assumption that the robustness of these measures is improved when they are gathered in controlled conditions. However, recent work in the laboratory and in daily life has emphasized that patterns of thought are intimately related to the context in which they emerge [5]. Since the tasks people perform in the lab are unlikely to perfectly match the activities people perform in daily life, the role that context plays in influencing thought patterns may be one reason why links between traits and patterns often do not generalize from the laboratory to daily life [6].

The influence that context has on thought patterns has been established both within the laboratory and in daily life (for a review see [7]). For example, Konu and colleagues [8] used a battery of laboratory-based activities, including cognitive and attentional tasks, videos, and audiobooks to establish that the patterns of thought people engage in change as a function of the tasks they engage in. In a similar vein, Mulholland and colleagues [9] established that in daily life, the thought patterns participants report vary with the activities they engage in. There were also similarities in how the ongoing context influenced the patterns that participants reported. In both studies, patterns of thought with (i) unpleasant, intrusive, and distracting features were identified that dominated low-demanding situations in the lab and daily life, (ii) thoughts with social and episodic features were important in social situations in daily life and tasks relying on social cognition in the lab, and (iii) patterns of detailed focus on a task were important during homework and while working in daily life, and were present during demanding laboratory task such as those depending on working memory. These prior lab and daily life studies [8,9] took advantage of a novel approach to measuring cognition known as multi-dimensional experience sampling (mDES, [10]). This approach asks participants to rate their experience along a number of dimensions (e.g., how detailed they are, whether are they intrusive, whether they involve other people, etc.) on multiple occasions. These data are then often decomposed into a set of underlying dimensions that describe the patterns of answers that the participants provided. The dimension derived from this decomposition creates a ‘thought space’ in which different experiential moments can be located [11–13]).

Emerging evidence suggests that one reason why mDES can characterize contextual influences on ongoing thought is because it provides high precision descriptions of individuals’ experiences. For example, states identified using mDES are associated with brain activity during attention tasks, where patterns of off-task thought with episodic features are related to greater activity within the medial prefrontal cortex, a region of the default mode network [14]. In contrast, patterns of detailed focus relating to a task are linked to greater activity in the frontoparietal network [12]. mDES is also sensitive to changes in brain activity during movie watching [11], highlighting that patterns of intrusive distraction are related to moments during a film when frontoparietal activity is reduced [11], a pattern consistent with the notion that intrusive thought reflects a failure to control cognition [15]. Recently, we demonstrated that mDES is sufficiently sensitive to variation in thought patterns to the extent that it can be used to build a fully generative model of the mapping between thought patterns and brain activity during task states [13].

### Current study

Together, these prior studies demonstrate that mDES provides a powerful tool for mapping thinking patterns in the lab and in daily life. Thus, our goal was to understand how the thought patterns mDES reveals in daily life relate to measures of mental health and well-being. Prior studies have already used experience sampling in daily life and in the laboratory to highlight important links between patterns of ongoing thought and factors that impact mental health and well-being, such as depression [16,17], anxiety [18], and obsessional thinking [19]. The goal of the current study was to leverage the precision mapping of experience sampling offered by mDES to understand the links between thought patterns in daily life and mental health and well-being. However, before examining links to mental health and well-being, we first investigated the reliability of the thought patterns produced using mDES. We took advantage of the fact that the thought patterns established by mDES are sensitive to the activities being performed in daily life [9] and examined whether the mapping between thought patterns and activities were consistent within the current dataset and also with those seen in our prior study [9]. Once we established the reliability of mDES as a tool for mapping thought patterns in daily life, we examined how the thought patterns established by our approach related to aspects of mental health and well-being as measured by a battery of questionnaires.

## Methods

### Ethics statement

This study was granted ethics clearance by the Queen’s University General Research Ethics Board. Participants gave informed, written consent through electronic documentation prior to taking part in research activities and were awarded two course credits and fully debriefed upon the completion of their participation.

### Participant population

A total of 261 participants (women = 227, men = 29, non-binary or similar gender identity = 4, preferred not to say = 1; *M* = 21.44; *SD* = 6.16; range = 17–52; note, two ages were recorded incorrectly and therefore not included and two additional ages were absent) were included in this study. Participants were recruited between January 9th, 2023, and April 10^th^, 2023, through the Queen’s University Psychology Participant Pool. Eligible participants were Queen’s University students enrolled in designated first- and second-year psychology courses.

### Procedure

Participants completed demographic questionnaires (i.e., age, gender, gender identity, sex assigned at birth, language(s) spoken most often at home, country of primary place of residence, program, and year of study). Participants also completed evidence-based health and well-being questionnaires, including the Quick Inventory of Depressive Symptomatology self-report (QIDS-SR_16_) [20], the Anxiety Sensitivity Index (ASI) [21], the Autism Spectrum Quotient (AQ) [22], the Adult ADHD Self-Report Scale (ASRS) [23], the Montgomery Asberg Depression Rating Scale self-report (MADRS-S) [24], the Overall Anxiety Severity and Impairment Scale (OASIS) [25], the Sheehan Disability Scale (SDS) [26], the MOS 36-item short form survey (SF-36) [27], and the World Health Organization Quality-of-Life Brief Version (WHOQOL-BREF) [28]. These questionnaires were selected based on a widespread, general approach to health and well-being, rather than clinical diagnosis. For the purpose of this study, our analysis was limited to the AQ, ASRS, MADRS-S, OASIS, and WHOQOL-BREF questionnaires, which were deemed the most relevant indicators of mental health and well-being. Once participants completed these questionnaires, they were notified via Samply Research, a mobile application, to complete mDES questionnaires eight times daily for five consecutive days between 7:00 am and 11:00 pm. Each questionnaire was randomly delivered within this 16-hour time window, with a minimum of 30 minutes in between each notification.

### Multi-dimensional experience sampling

Participants received notifications on their phones through the Samply Research mobile application. All responses were made in reference to their thoughts, feelings, social environment, location, and activity immediately before receiving the notification. This study used a 16-question mDES battery that has been used in prior studies [11,13] (see Table 1). The mDES questions were always asked first and delivered in a random order. Participants then rated their stress on a 1-to-10 Likert scale. For the purpose of this study, responses to the stress question were not analyzed. Next, participants answered questions about their physical and virtual social environments (Table 2). Finally, participants indicated the type of physical location they were in (Table 3) and their primary activity (Table 4). The list of activities given to participants was developed from the Day Reconstruction Method [29] and modified based on the available options in our prior studies [9,30].

**Table 1:** mDES questions.

Dimension	Question	Scale low	Scale high
Task	My thoughts were focused on an external task or activity:	Not at all	Completely
Future	My thoughts involved future events:	Not at all	Completely
Past	My thoughts involved past events:	Not at all	Completely
Self	My thoughts involved myself:	Not at all	Completely
People	My thoughts involved other people:	Not at all	Completely
Emotion	The emotion of my thoughts was:	Negative	Positive
Images	My thoughts were in the form of images:	Not at all	Completely
Words	My thoughts were in the form of words:	Not at all	Completely
Sounds	My thoughts were in the form of sounds:	Not at all	Completely
Detailed	My thoughts were detailed and specific:	Not at all	Completely
Deliberate	My thoughts were:	Spontaneous	Deliberate
Problem	I was thinking about solutions to problems or goals:	Not at all	Completely
Intrusive	My thoughts were intrusive:	Not at all	Completely
Knowledge	My thoughts contained information I already knew (e.g., knowledge or memories):	Not at all	Completely
Absorption	I was absorbed in the contents of my thoughts:	Not at all	Completely
Distracting	My thoughts were distracting me from what I was doing:	Not at all	Completely

Participants rated statements on a 1-to-10 Likert scale.

**Table 2:** Social environment questions.

Environment	Question	Environment type
Physical	Were you alone, or physically with other people?	Alone
		Around people but not interacting with them
		Around people and interacting with them
	Where you alone, or physically with a pet?	Not with a pet
		Around a pet but not interacting with them
		Around a pet and interacting with them
Virtual	Were you alone, or virtually with other people?	Alone
		Around people but not interacting with them (e.g., reading messages but not replying, being on a video call but not participating, etc.)
		Around people and interacting with them (e.g., direct communication with another person by text, instant messaging, calling, video calling, etc.)

**Table 3:** Physical location question.

List type	Question	Location list
Location	Where were you?	Inside a home
		Inside a shop
		Inside a workplace
		Inside (other)
		Outside in a city or town
		Outside in nature
		Outside (other)

If participants selected ‘Inside (other)’, or ‘Outside (other)’, they were asked to specify their location.

**Table 4:** Primary activity question.

List type	Question	Activity list
Activity	What were you doing?	Eating
		Homework
		Household chores
		Listening to music
		Napping or resting
		Nothing or waiting
		Personal exercise
		Personal hygiene care
		Physical leisure or sports
		Reading
		Shopping
		Talking in person
		Talking on the phone
		Video-calling
		Messaging by phone/device
		Traveling or commuting
		Using a computer or an electronic device
		Walking the dog
		Watching TV
		Working
		Other activity

If participants selected ‘Other Activity’, they were asked to specify what they were doing. If participants selected ‘Using a computer or an electronic device’, they were asked to further specify their activity from a list of alternative activities that included ‘Social media: Passive scrolling’, ‘Social media: Active engagement’, ‘Gaming’, ‘Admin (e.g., banking, calendar, etc.)’, and ‘Other’. If participants selected ‘Other’, they were asked to further specify what they were doing.

## Analysis

### Data and code availability statement

All custom code used to prepare data for analysis and figure development is available online at https://github.com/ThinCLabQueens and https://github.com/BridgMul10. Anonymized data has been uploaded to a publicly accessible database, Figshare, and is available online at https://doi.org/10.6084/m9.figshare.28851896.

### Principal Component Analysis: mDES questions

**Fig 1:**
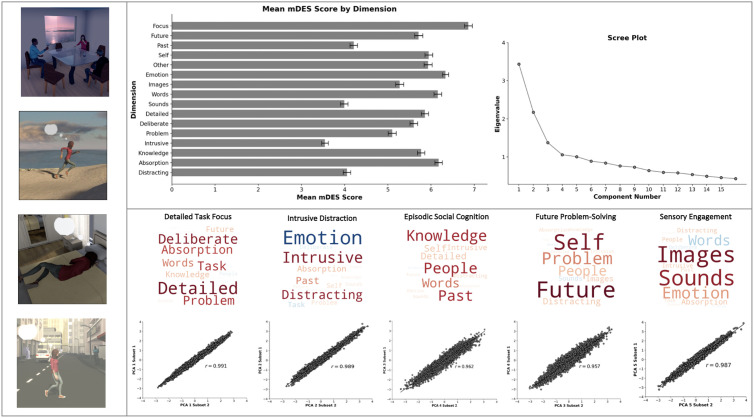
The within-dataset consistency of the dimensional structure of ongoing experience in daily life. Upper horizontal panel: (Left) Bar graph describing the mean mDES score for each dimension. Error bars represent 99% confidence intervals. (Right) Scree plot generated from PCA of mDES data. Lower horizontal panel: Word clouds describing the experiential features that contribute the most to the dimensions of experience as described by PCA. Larger fonts describe features with more importance on the dimension (i.e., stronger loadings) and the font colour denotes direction of the loading (i.e., warm colours relate to positive loadings). For the purpose of exposition, these components are named based on their strongest features. From left to right: ‘Detailed Task Focus’, ‘Intrusive Distraction’, ‘Episodic Social Cognition’, ‘Future Problem-Solving’, and ‘Sensory Engagement’. Scatter plots below show the split-half reliability of these dimensions (y-axis: subset 1; x-axis: subset 2). Left vertical panel: Illustrations of different activities in daily life.

As in our prior studies (e.g., [8,9,12,14,30]), common patterns of thought (Fig 1) were identified by applying Principal Component Analysis (PCA) using varimax rotation to the data generated from the 16 mDES questions (Table 1) using IBM SPSS Statistics (version 29). PCA was applied at the observation level and included 6776 observations. The loadings from the five components with an eigenvalue > 1 were retained for further analysis (see Table 5 and Fig 1).

**Table 5:** PCA loadings generated from mDES questions.

Dimension	‘Detailed Task Focus’	‘Intrusive Distraction’	‘Episodic Social Cognition’	‘Future Problem-Solving’	‘Sensory Engagement’
Task	.605	-.207	.017	-.033	.142
Future	.228	.068	.106	.797	.064
Past	-.021	.408	.636	.054	.169
Self	.003	.166	.227	.730	-.009
People	-.079	-.004	.645	.253	.208
Emotion	.018	-.730	.115	.042	.351
Images	.017	.048	.032	.216	.742
Words	.435	.020	.452	-.092	-.264
Sounds	.051	.080	.127	-.177	.680
Detailed	.748	.038	.224	.102	.034
Deliberate	.702	-.109	-.059	.009	-0.137
Problem	.609	.146	.010	.412	-0.052
Intrusive	-.013	.710	.211	.137	0.207
Knowledge	.240	.059	.651	.141	-.002
Absorption	.560	.267	.084	.155	.230
Distracting	-.015	.689	.174	.220	.228

### Reliability analysis: mDES data

Component reliability analysis was conducted in IBM SPSS Statistics (version 29). mDES data was randomly shuffled and divided into two halves (n = 3445 probes per sample). PCA using varimax rotation was applied to each subset separately and Pearson correlation was run to compare the component loadings generated from each subset with the overall solution. The higher the correlation between the two halves of the data to the overall sample, the more consistent the dimensional structure within the overall sample was.

### Linear mixed modeling: Thought-activity mapping

**Fig 2:**
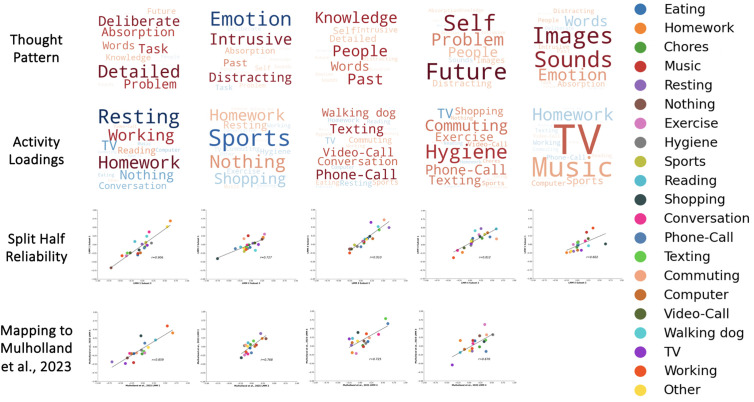
Consistency of thought-activity mapping. First row: Thought patterns. Words represent PCA loadings for mDES data and LMM loadings for activities. Larger fonts are features with more importance (i.e., higher loadings) and colour denotes features with a similar loading on the dimensions (i.e., warm colours relate to positive loadings). See Tables 5–10 for specific component loadings. Second row: Activity loadings. In these word clouds, the larger the font, the stronger the activity loads onto that thought component and activities with a similar colour load in the same direction. Third row: Scatter plots showing the within-dataset study consistency of the thought-activity mapping (y-axis: activity loadings from subset 1; x-axis: activity loadings from subset 2). Fourth row: scatter plots showing the cross-dataset consistency of the thought-activity mapping (y-axis: activity loadings from Mulholland et al. [9]; x-axis: within-dataset activity loadings).

**Fig 3:**
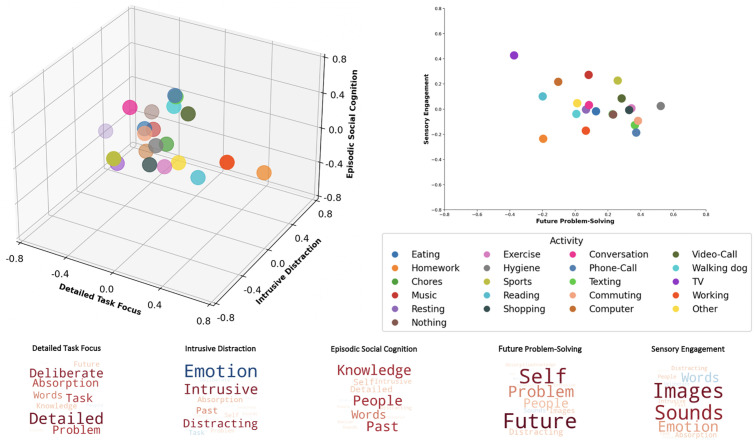
The multivariate landscape of thought-activity mapping in daily life. The thought-activity mapping revealed by our analysis is presented in two- and three-dimensional spaces to provide the opportunity to visualize the multivariate nature of the relationships. Left image: Thought-activity mapping in a three-dimensional thought space. Each point is an activity. The x-axis represents the activity loadings for ‘Detailed Task Focus’, the y-axis represents the activity loadings for ‘Intrusive Distraction’, and the z-axis represents the activity loadings for ‘Episodic Social Cognition’. Right image: Thought-activity mapping in a two-dimensional thought-space. The y-axis represents activity loadings for ‘Sensory Engagement’ and the x-axis represents the activity loadings for ‘Future Problem-Solving’. Lower panel: This shows the feature loadings for each dimension of the thought-space in the form of word clouds. As described previously, larger fonts are activities with more importance (i.e., higher loadings on a dimension) and colour denotes direction (e.g., warm colours relate to positive loadings).

To analyze contextual distributions of thought in relation to activities, we conducted a series of linear mixed models (LMMs), one with each thought component as the dependent variable and activity as the independent variable, to examine whether patterns of thought varied across activity categories. Restricted maximum likelihood (REML) was used as the estimation method and a variance components model was used as the covariance type. Participants were included as a random intercept. The parameter estimates for each activity in each model were saved for the eventual generation of activity word clouds that describe the experiential features that contribute to each dimension (Fig 2). This analysis is identical to that found in Mulholland et al. [9]. The parameter estimates were also saved to be used in reliability analysis (Fig 3).

**Table 10:** LMM estimated marginal means for the ‘Sensory Engagement’ component.

Primary activity	Mean	Std. error	Lower bound 95% confidence interval	Upper bound 95% confidence interval
Eating	-0.017	0.049	-0.113	0.080
Homework	-0.235	0.041	-0.315	-0.155
Chores	-0.041	0.061	-0.160	0.079
Music	0.272	0.065	0.145	0.400
Resting	-0.003	0.049	-0.098	0.092
Nothing	-0.043	0.060	-0.161	0.075
Exercise	0.007	0.081	-0.152	0.165
Hygiene	0.025	0.063	-0.098	0.148
Sports	0.227	0.125	-0.018	0.471
Reading	0.101	0.081	-0.059	0.260
Shopping	-0.007	0.094	-0.191	0.177
Conversation	0.032	0.048	-0.063	0.126
Phone-call	-0.186	0.101	-0.385	0.013
Video-call	0.085	0.090	-0.091	0.261
Texting	-0.127	0.085	-0.293	0.039
Commuting	-0.093	0.079	-0.248	0.061
Computer	0.217	0.048	0.123	0.311
Walking dog	-0.038	0.162	-0.355	0.279
TV	0.427	0.048	0.333	0.520
Working	-0.171	0.057	-0.282	-0.060
Other	0.047	0.049	-0.048	0.143

**Table 6:** LMM estimated marginal means for the ‘Detailed Task Focus’ component.

Primary activity	Mean	Std. error	Lower bound 95% confidence interval	Upper bound 95% confidence interval
Eating	-.198	0.044	-.284	-.112
Homework	.664	.033	.599	.729
Chores	-.017	.058	-.130	.096
Music	-.205	.062	-.326	-.083
Resting	-.686	.043	-.770	-.602
Nothing	-.344	.057	-.455	-.233
Exercise	.042	.080	-.114	.199
Hygiene	-.029	.060	-.146	.088
Sports	-.149	.127	-.398	.099
Reading	.281	.080	.124	.438
Shopping	-.042	.094	-.226	.143
Conversation	-.293	.043	-.377	-.209
Phone-call	.043	.102	-.157	.243
Video-call	.168	.089	-.007	.344
Texting	.025	.084	-.140	.189
Commuting	-.139	.077	-.291	.012
Computer	-.250	.042	-.333	-.167
Walking dog	-.005	.165	-.329	.318
TV	-.388	.042	-.470	-.306
Working	.544	.052	.441	.646
Other	.122	.043	.038	.206

**Table 7:** LMM estimated marginal means for the ‘Intrusive Distraction’ component.

Primary activity	Mean	Std. error	Lower bound 95% confidence interval	Upper bound 95% confidence interval
Eating	-.036	.048	-.130	.059
Homework	.232	.038	.157	.308
Chores	0.026	.062	-.147	.095
Music	.102	.066	-.028	.232
Resting	.199	.048	-.106	.292
Nothing	.316	.061	.197	.436
Exercise	-.156	.084	-.320	.009
Hygiene	-.160	.064	-.285	-.035
Sports	-.552	.131	-.810	-.295
Reading	-.079	.084	-.244	.086
Shopping	-.229	.098	-.422	-.037
Conversation	-.084	.047	-.177	.009
Phone-call	-.006	.106	-.214	.202
Video-call	-.031	.093	-.214	.152
Texting	.041	.088	-.131	.214
Commuting	-.133	.081	-.293	.026
Computer	.070	.047	-.022	.162
Walking dog	.063	.171	-.272	.397
TV	-.121	.047	-.213	-.030
Working	-.125	.057	-.236	-.014
Other	-.090	.043	-.183	.004

**Table 8:** LMM estimated marginal means for the ‘Episodic Social Cognition’ component.

Primary activity	Mean	Std. error	Lower bound 95% confidence interval	Upper bound 95% confidence interval
Eating	0.170	0.047	0.078	0.262
Homework	-0.178	0.036	-0.247	-0.108
Chores	0.057	0.062	-0.063	0.178
Music	0.070	0.066	-0.060	0.200
Resting	-0.194	0.046	-0.284	-0.104
Nothing	0.082	0.061	-0.036	0.201
Exercise	-0.089	0.085	-0.256	0.078
Hygiene	0.124	0.064	-0.001	0.249
Sports	0.195	0.135	-0.070	0.459
Reading	-0.171	0.085	-0.338	-0.003
Shopping	-0.050	0.100	-0.246	0.147
Conversation	0.405	0.046	0.315	0.494
Phone-call	0.610	0.109	0.397	0.823
Video-call	0.469	0.095	0.283	0.656
Texting	0.561	0.089	0.385	0.736
Commuting	0.202	0.083	0.040	0.364
Computer	-0.178	0.045	-0.267	-0.089
Walking dog	0.429	0.176	0.085	0.773
TV	-0.243	0.045	-0.331	-0.155
Working	0.136	0.056	0.026	0.245
Other	-0.058	0.046	-0.148	0.032

**Table 9:** LMM estimated marginal means for the ‘Future Problem-Solving’ component.

Primary activity	Mean	Std. error	Lower bound 95% confidence interval	Upper bound 95% confidence interval
Eating	.127	0.047	0.035	0.218
Homework	-0.196	0.035	-0.265	-0.128
Chores	0.229	0.062	0.108	0.349
Music	0.082	0.066	-0.048	0.213
Resting	0.066	0.046	-0.023	0.156
Nothing	0.232	0.061	0.113	0.351
Exercise	0.344	0.086	0.176	0.512
Hygiene	0.523	0.064	0.398	0.648
Sports	0.260	0.136	-0.007	0.528
Reading	-0.199	0.086	-0.367	-0.030
Shopping	0.330	0.101	0.132	0.529
Conversation	0.084	0.045	-0.005	0.174
Phone-call	0.373	0.110	0.158	0.588
Video-call	0.284	0.096	0.096	0.473
Texting	0.365	0.090	0.188	0.541
Commuting	0.384	0.083	0.221	0.546
Computer	-0.104	0.045	-0.192	-0.015
Walking dog	0.007	0.177	-0.340	0.355
TV	-0.374	0.045	-0.461	-0.286
Working	0.065	0.056	-0.044	0.175
Other	0.012	0.046	-0.078	0.101

### Reliability analysis: Within-dataset consistency of thought-activity mapping

Activity reliability analysis was conducted in IBM SPSS Statistics (version 29). As before, component scores were split into two subsets (n = 3445 probes per subset). We conducted a series of LMMs, with thought components for each subset as the dependent variable and activity as the independent variable. REML was used as the estimation method and a variance component model was used as the covariance type. Participants were included as a random intercept. Pearson correlation was run on the parameter estimates generated from each subset for each activity. This analysis allowed us to estimate whether the results of our whole sample LMM were generalizable to subsets of the data.

### Reliability analysis: Thought-activity mapping cross-dataset comparison

Reliability analysis was conducted in IBM SPSS Statistics (version 29). The LMM parameter estimates from Mulholland et al. [9] contained a sample of n = 1451 probes, and the LMM parameter estimates from our current study contained a sample of n = 6889 probes. Pearson correlation was run on the parameter estimates for overlapping activities from each dataset. This analysis allowed us to estimate whether the results of our whole sample LMM in the current dataset were generalizable to those seen in the prior dataset.

### Linear mixed modeling: Social environment data

To analyze contextual distributions of thought in relation to socialization, we conducted a series of LMMs, one with each thought component as the dependent variable and social environment as the independent variable each type of physical or virtual. These analyses allowed us to examine whether patterns of thought varied meaningfully across social environments. REMLwas used as the estimation method and a variance components model was used as the covariance type. Participants were included as a random intercept.

### Multiple regression: Linking mDES to traits

To analyze whether mDES can differentiate individuals based on underlying traits, we performed a series of multiple regressions with average PCA component scores for each individual in the overall thought space as the dependent variable and mental health and well-being trait questionnaire scores as explanatory variables. Trait questionnaire scores included in this analysis were the AQ, ASRS, MADRS, OASIS, and WHOQOL-BREF, all of which were z-scored. The WHOQOL-BREF questionnaire was subdivided into four categories based on scoring domains: physical health, psychological health, social relationships, and environment. mDES data was reduced to a single score for each participant.

## Results

### Patterns of ongoing thought

First, mean scores for each mDES dimension were calculated and are shown in the bar graph in Fig 1. Next, as is standard in our laboratory work (e.g., [8,12,14,30]),the mDES data was decomposed using PCA with varimax rotation to reveal dimensions that best described the patterns of ongoing thought reported by participants. Based on eigenvalue > 1, five components were selected for further analysis (see Fig 1 for scree plot). PCA loadings (Table 5) from the five components were used to generate thought word clouds (Fig 1). Components were named based on mDES dimensions that dominated their composition. Component 1 (21.5% of variance) was labelled ‘Detailed Task Focus’ because loadings were high for ‘detailed’, ‘deliberate’, ‘problem’, and ‘task’ (Fig 1). Component 2 (13.6% of variance) was labelled ‘Intrusive Distraction’ because loadings were high for ‘(negative) emotion’, ‘intrusive’, and ‘distracting’ (Fig 1). Component 3 (8.6% of variance) was labelled ‘Episodic Social Cognition’ because loadings were high for dimensions such as ‘knowledge’, ‘person’, and ‘past’ (Fig 1). Component 4 (6.6% of variance) was labelled ‘Future Problem-Solving’ because loadings were high for dimensions such as ‘future’, ‘self’, and ‘problem’ [as in problem-solving] (Fig 1). Component 5 (6.3% of variance) was labelled ‘Sensory Engagement’ because loadings were high for dimensions such as ‘images’ and ‘sounds’ (Fig 1). Please note that these terms are used for convenience to summarize the features that characterize each component. They do not constitute the only labels which could be applied to these patterns.

### Component reliability

To understand the robustness of the dimensions within our sample, we conducted a split-half reliability analysis. In this analysis, the data was divided into two random samples (n = 3445 probes per sample) and then examined to determine how the components generated in each half of the data related to each other. We used the robustness of the solutions across PCAs with 3-, 4-, and 5-component solutions as a complementary method to determine the best solution for the entire sample. The mean correlation for the set of the homologous pairs from each solution was calculated, with a higher score reflecting the most reproducible components. The 3-, 4-, and 5-component solutions all produced highly reliable results, with average homologue similarities scores of *r* = .996 (3-component solution, range *r *= .994-.995, Fig 1), *r *= .93525 (4-component solution, range *r *= .869-.978, Fig 1), and *r *= 0.9772 (5-component solution, range *r *= .957-.991, Fig 1). Since similarity was high for 3-, 4-, and 5-component solutions, we focused our analysis on the 5-component solution.

### Thought-activity mapping

Having identified the stability of the dimensions identified within this dataset, we examined if mDES provides a reliable way to map cognition in daily life onto activities that are being performed. To this end, we ran a series of LMMs in which the activities were the explanatory variables and the mDES scores for each dimension from the common thought space were the dependent variables. In each case we found a significant association between reported patterns of thought and activities (‘Detailed Task Focus’ (*F*(21, 6639.50) = 74.08, *p* < .001); ‘Intrusive Distraction’ (*F*(21, 6591.47) = 10.21, *p* < .001); ‘Episodic Social Cognition’ (*F*(21, 6632.08) = 19.08, *p* < .001); ‘Future Problem-Solving’ (*F*(21, 6648.67) = 18.26, *p *< .001); ‘Sensory Engagement’ (*F*(21, 6559.04) = 16.82, *p* < .001)). To visualize the thought-activity mapping, we generated a set of word clouds based on the estimated marginal means for reported activities in each component (Fig 2). ‘Detailed Task Focus’ was high when doing homework and working and lowest when resting, watching TV, and doing nothing. ‘Intrusive Distraction’ was highest when doing nothing, resting, and doing homework, and lowest when playing sports and shopping. ‘Episodic Social Cognition’ features were highest when talking on the phone, texting, video-calling, walking the dog, talking in person, commuting, and playing sports and lowest when watching TV, resting, using a computer, and doing homework. ‘Future Problem-Solving’ was highest during hygiene activities, texting, commuting, talking on the phone, exercise, and shopping and lowest when watching TV, reading, and doing homework. Lastly, ‘Sensory Engagement’ had high loadings when watching TV, listening to music, playing sports, and using a computer and low loadings when doing homework, talking on the phone, and working.

Next, we investigated the robustness of the thought-activity mapping produced by mDES. Our first analysis examined the consistency of the mapping within the current data by performing a split-half reliability analysis. We created two subsets of our data (n = 3445 probes per subset). In each case, we found a significant association between each of the reported thought patterns and activities in each subset (‘Detailed Task Focus’ subset 1 (*F*(21, 3284.10) = 36.96, *p* < .001) and subset 2 (*F*(21, 3333.340) = 36.101, *p* < .001); ‘Intrusive Distraction’ subset 1 (*F*(21, 3274.05) = 4.63, *p* < .001) and subset 2 (*F*(21, 3285.57) = 6.53, *p* < .001); ‘Episodic Social Cognition’ subset 1 (*F*(21, 3323.14) = 8.92, *p* < .001) and subset 2 (*F*(21, 3271.06) = 10.32, *p* < .001); ‘Future Problem-Solving’ (*F*(21, 3285.56) = 10.02, *p* < .001) and subset 2 (*F*(21, 3294.73) = 9.06, *p* < .001); ‘Sensory Engagement’ subset 1 (*F*(21, 3242.37) = 9.69, *p* < .001) and subset 2 (*F*(21, 3239.06) = 8.13, *p* < .001)).

To understand whether the thought-activity mapping in daily life was similar for each subset of the data, the loadings of each activity on each component was correlated (see Methods). Correlations between the LMM activity estimated marginal means in each subset ranged from *r *= .60-.91, with the highest reproducibility for the ‘Detail Task Focus’ component and the lowest for the ‘Sensory Engagement’ component. This shows a reasonably high degree of consistency in the feature loadings for each dimension of experience within the current sample.

Next, we examined how the thought-activity mapping from the current study mapped onto those from the homologous components from Mulholland et al. [9]. To this end, we used Pearson correlation to compare the thought-activity mapping from our prior study to the thought-activity mapping found in our omnibus sample. Mulholland et al. [9] did not include a ‘Sensory Engagement’ component (because the modality questions did not include sounds), so only four components were compared. This analysis identified correlations with a range of *r *= .67-.86, with the strongest thought-activity mapping including ‘Detailed Task Focus’ and the lowest including ‘Future Problem-Solving’. This analysis shows a reasonably high degree of overlap between the thought-activity mapping as described in these data and those seen in our prior study [9].

One feature of our analysis is that it allows us to understand experience within each activity as a linear combination of different thought components, which can be represented graphically by the location of an activity in a multivariate thought space. To visualize the data this way, the estimated marginal means for each activity derived from the LMMs were plotted against each mDES component in the common thought space. We present these data in a three-dimensional space constructed using the three most reliable components (i.e., ‘Detailed Task Focus’, ‘Intrusive Distraction’, and ‘Episodic Social Cognition’) as the dimensions. We also present a two-dimensional space that describes the relationship between activities in terms of their weighting for ‘Future Problem-Solving’ and ‘Sensory Engagement’. These are both presented in Fig 3, which show how certain activities occupy extreme values on multiple components. For example, ‘homework’ is high on both the ‘Detailed Task Focus’ and ‘Intrusive Distraction’ components but low on the ‘Episodic Social Cognition’, ‘Future Problem-Solving’, and ‘Sensory Engagement’ components.

### mDES thought dimensions and social environments

Another way to examine the robustness of mDES as a tool for mapping cognition in daily life is by measuring the similarity in how the thought patterns map onto social environments in daily life. Prior studies (e.g., [9]) have found that being alone compared to being with other people has important implications for a person’s thought patterns, particularily with facilitating patterns of thought with social and episodic features. We ran a series of LMMs in which different social environments (Table 2) were the explanatory variable and the mDES scores from the common thought space were the dependent variables. We found a significant association between reported patterns of thought and physical social environments for each component. ‘Detailed Task Focus’ (*F*(3, 6721.87) = 16.06, *p* < .001) was lowest when with people and interacting with them (*M* = -.08, 95% CI [-.08, -.13]) and highest when with people but not interacting with them (*M* = .14, 95% CI [.08, -.21]). ‘Intrusive Distraction’ (*F*(3, 6656.39) = 37.54, *p* < .001) was lowest when with people and interacting with them (*M* = -.15, 95% CI [-.22, -.08]) and highest when alone (*M* = .11, 95% CI [.05, .18]). ‘Episodic Social Cognition’ (*F*(3, 6699.28) = 56.29, *p* < .001) was highest when with people and interacting with them (*M* = .22, 95% CI [.16, .28]) and lowest when with people but not interacting with them (*M* = -.15, 95% CI [-.08, -.22]). ‘Future Problem-Solving’ (*F*(3, 6716.79) = 2.79, *p* = .039) was lowest when around people and interacting with them (*M* = -0.46, 95% CI [-.11, .02]) and highest when around people but not interacting with them (*M* = .04, 95% CI [-.02, .15]). Finally, ‘Sensory Engagement’ (*F*(3, 6619.47) = 5.30, *p* = .001) was highest when alone (*M* = .03, 95% CI [-.08, .02]). A broadly similar pattern was found for our analysis of the consequence of virtual environments. ‘Detailed Task Focus’ (*F*(3, 6705.72) = 2.67, *p* = .046) was highest when with people but not interacting with them (*M* = .05, 95% CI [-.03, .14]) and lowest when alone (*M* = -.03, 95% CI [-.83, .02]). ‘Intrusive Distraction’ (*F*(3, 6695.70) = 11.53, *p *< .001) was least prevalent when participants were with people and interacting with them (*M* = -.11, 95% CI [-.19, -.03]) and most prevelant when around people but not interacting with them (*M* = .07, 95% CI [-.02, .16]). ‘Episodic Social Cognition’ (*F*(3, 6715.54) = 39.37, *p* < .001) was highest when around people and interacting with them (*M* = .27, 95% CI [.19, .34]) and lowest when alone (*M* = -.07, 95% CI [.19, .33]). ‘Future Problem-Solving’ (*F*(3, 6709.60) 9.55, *p *< .001) was highest when around people and interacting with them (*M* = .15, 95% CI [.07, .22]) and lowest when around people but not interacting with them (*M* = -.03, 95% CI [-.12, .05]). Finally, ‘Sensory Engagement’ (*F*(3, 6663.16) = 2.89, *p* = .03) was highest when alone (*M* = .01, 95% CI [-.05, .08]) and lowest around people but not interacting with them (*M* = -.003, 95% CI [-.09, .09]). These analyses replicate prior observations that found a mapping of social cognition onto social situations (e.g., [9,30]) but extends this to establish that such situations also reduce intrusive features of cognition.

### mDES thought dimensions and traits

**Fig 4:**
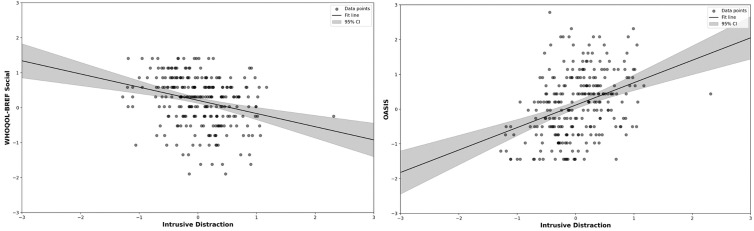
mDES and Trait Multiple Regression Results. Left panel: Scatterplot with WHOQOL-BREF social relationships on the y-axis and ‘Intrusive Distraction’ PCA component scores on the x-axis. Right panel: Scatterplot with OASIS on the y-axis and ‘Intrusive Distraction’ PCA component scores on the x-axis. For both scatterplots, all values were mean z-scored per participant.

Having established a high level of consistency between the thought-activity mapping seen in the current study and prior studies [9,30], the final goal of our study was to investigate whether mDES thought dimensions can differentiate people based on traits associated with mental health and well-being. To do so, we performed a series of multiple regressions with the average loading of each individual on each dimension of the common thought space as the dependent variables and their scores on the trait questionnaires as explanatory variables. Results indicated the location of each individual on the ‘Intrusive Distraction’ dimension (*F*(8, 252) = 8.35, *p* = < .001; see Table 11 for results for each component) could be predicted based on high levels of anxiety (Beta = 0.23, *p* = .003) and low levels of social well-being (Beta = -0.17, *p* = .010). This analysis, therefore, shows a clear mapping between patterns of ‘Intrusive Distraction’ with higher levels of anxiety and lower social well-being (Fig 4).

**Table 11:** Mental Health and Well-Being Multiple Regression Results Per PCA Component Scores.

Component	Model	Sum of squares	df	Mean square	F	Sig.
Detailed Task Focus	Regression	.660	8	.083	.444	.894
	Residual	46.866	252	.186		
	Total	47.526	260			
Intrusive Distraction	Regression	15.071	8	1.884	8.348	<.001
	Residual	56.869	252	.226		
	Total	71.940	260			
Episodic Social Cognition	Regression	2.127	8	.266	1.085	.374
	Residual	61.773	252	.245		
	Total	63.900	260			
Future Problem-Solving	Regression	3.129	8	.391	1.806	.076
	Residual	54.569	252	.217		
	Total	57.698	260			
Sensory Engagement	Regression	1.248	8	.156	.415	.911
	Residual	94.767	252	.376		
	Total	96.015	260			

## Discussion

Our study sought to investigate the underlying relationships between patterns of reported thought, activity, and measures of mental health and well-being in daily life. First, we investigated the stability of the patterns of thoughts reported in our study, establishing a reasonable range of consistency for a five component solution. These components were ‘Detailed Task Focus’, ‘Intrusive Distraction’, ‘Episodic Social Cognition’, ‘Future Problem-solving’, and ‘Sensory Engagement’. Second, we examined the reproducibility of how activities in daily life map onto these dimensions. The thought-activity mapping was both consistent within these data and were similar to the thought patterns described by Mulholland et al. [9] (Fig 2), who administered mDES in daily life to a different sample of participants. We also replicated patterns found in prior studies that show, for example, that patterns of ‘Episodic Social Cognition’ are higher when individuals are interacting either physically or virtually [9], or were reduced during COVID-19 lockdowns in the United Kingdom due to reductions in opportunities for socialization [30]. Taken together, these analyses show that the thought space generated by the application of mDES to daily life provides a reproducible measure of ongoing thought that organizes the activities we engage in as we go about our daily lives in a meaningful manner (see Fig 3). It is important to note that populations in both studies are undergraduate students, so this reproducibility highlights that if mDES is employed within a similar group of individuals, then it captures thought patterns that are reasonably consistent in their profiles and their associations with activities. Further studies are needed to understand how thought-activity mapping varies across ages and cultures, as it is possible that the observed thought patterns, as well as their links to activities or social contexts, could conceivably change if they were measured in different cultures or age groups.

### Links between thought patterns in daily life and psychological well-being

The main goal of our study was to understand the links between patterns of thought in daily life and mental health and well-being. We found that patterns of thought that loaded heavily on ‘Intrusive Distraction’ were associated with greater levels of anxiety and less social support (Fig 4). Importantly, this thought pattern showed a reasonable degree of consistency in terms of how it mapped onto activities within this sample (*r *= .77) and with our prior study (*r* = .73), highlighting that this thought component has a reasonably consistent thought-activity mapping.

Contemporary perspectives that emphasize patterns of thought often have an important social component (e.g., [31]). One important contribution of our study is that our analysis suggests that higher levels of ‘Intrusive Distraction’ are also linked to social processes. As well as linking intrusive thought to relatively low social well-being, our analysis also shows that this pattern of thought tends to dominate solo activities (such as exercise, doing nothing, and homework) and is relatively absent during social interactions, either virtually or in person. Interestingly, despite intrusive thought being relatively high during exercise, this component was relatively low during sports, providing further evidence that social contexts may reduce levels of unpleasant intrusive experiences. Importantly, these data are consistent with prior studies that show links between anxiety and increased levels of mind-wandering (e.g., [32]), a self-generated state that is likely to have distracting features [15,33]. Altogether, therefore, these data suggest that unpleasant intrusive thoughts may be promoted by loneliness and social isolation, a situation that may promote personal worries, often known as current concerns [34]. It is possible based on our study that interventions that increase opportunities for socialization may be an important way to help individuals who suffer from anxiety.

### Open questions

As well as detailing links between thinking in daily life and mental health and well-being, our study also raises a number of important open questions. For example, the consistency of the thought-activity mapping suggests that in any given population there may be systematic relationships between what people think and what they do in daily life. Based on these results, it may be possible to develop an understanding of the activities in daily life that are most related to different features of a person’s thought content, a taxonomy of mapping between thoughts and activities which, as a discipline, we currently lack. It is worth noting that the capacity for mDES to map thoughts in the lab and in daily life [35] makes it a useful tool for this goal. The relative ease with which we can administer mDES in daily life makes it possible to study a relatively large and socioculturally diverse population (e.g., older adults or individuals from a different culture). Thus, while our study shows that mDES is reliable, the specific results presented in this study may not generalize to different cultures, or ages. Our methodology of smartphone sampling is free and easy to use, so future studies can address this gap by broadening the scope of their sampling population in a reasonably accessible manner. It is also important to note that there were a number of instances of incorrect and/or missing demographic data in our data which should be considered a limitation of our current study.

It is also likely that the battery of questions we used in our study could be improved. We used a combination of mDES questions that had been used previously in both lab and daily-life settings, as well as new mDES questions based on previously identified limitations. Mulholland et al. [9] noted that including a single modality question does not allow participants to describe their conscious experience of an activity fully. In that study, a component that was common while listening to music was dominated by thoughts with images as a key feature. Notably, in that mDES battery there were no questions about sounds. In light of this discovery, we decided to replace the modality probe with three questions relating to an individual’s sensory experience (i.e., images, words, and sounds). The inclusion of these three questions most likely led to the discovery of the ‘Sensory Engagement’ component in our current study. It is important to note that a similar component was observed in the context of movie watching, using the same set of items applied in the current study [11], and was associated with periods of films when brain activity was activated within sensory systems (both auditory and visual). This demonstrates that it is possible to optimize the questions included in mDES approaches that could, in the future, provide more accurate ways for participants to describe their conscious experience. In the same vein, there are likely to be ways to improve the measures of mental health and well-being we employed in this analysis. The health and well-being questionnaires were selected based on a widespread, general approach to health and well-being, rather than clinical diagnosis. In the future, more accurate or informative questionnaires could be selected that would provide a better understanding of how our thoughts in daily life impact upon our mental health and well-being.

## References

[1] Asztalos M, Wijndaele K, De Bourdeaudhuij I, Philippaerts R, Matton L, Duvigneaud N, et al. Specific associations between types of physical activity and components of mental health. J Sci Med Sport. 2009;12(4):468–74. doi: 10.1016/j.jsams.2008.06.009 18768366

[2] Kucyi A, Kam JWY, Andrews-Hanna JR, Christoff K, Whitfield-Gabrieli S. Recent advances in the neuroscience of spontaneous and off-task thought: implications for mental health. Nat Ment Health. 2023;1(11):827–40. doi: 10.1038/s44220-023-00133-w 37974566 PMC10653280

[3] Vatansever D, Karapanagiotidis T, Margulies D, Jefferies E, Smallwood J. Distinct patterns of thought mediate the link between brain functional connectomes and well-being. Netw Neurosci. 2020;4(3):637–57. 32885119 10.1162/netn_a_00137 PMC7462429

[4] Kane MJ, Brown LH, McVay JC, Silvia PJ, Myin-Germeys I, Kwapil TR. For whom the mind wanders, and when: an experience-sampling study of working memory and executive control in daily life. Psychol Sci. 2007;18(7):614–21. doi: 10.1111/j.1467-9280.2007.01948.x 17614870

[5] Smallwood J, Turnbull A, Wang H-T, Ho NSP, Poerio GL, Karapanagiotidis T, et al. The neural correlates of ongoing conscious thought. iScience. 2021;24(3):102132. doi: 10.1016/j.isci.2021.102132 33665553 PMC7907463

[6] Kane MJ, Gross GM, Chun CA, Smeekens BA, Meier ME, Silvia PJ, et al. For Whom the Mind Wanders, and When, Varies Across Laboratory and Daily-Life Settings. Psychol Sci. 2017;28(9):1271–89. doi: 10.1177/0956797617706086 28719760 PMC5591044

[7] Turnbull A, Poerio GL, Ho NS, Martinon LM, Riby LM, Lin FV, et al. Age-related changes in ongoing thought relate to external context and individual cognition. Conscious Cogn. 2021;96:103226. doi: 10.1016/j.concog.2021.103226 34689074

[8] Konu D, Mckeown B, Turnbull A, Siu Ping Ho N, Karapanagiotidis T, Vanderwal T, et al. Exploring patterns of ongoing thought under naturalistic and conventional task-based conditions. Conscious Cogn. 2021;93:103139. doi: 10.1016/j.concog.2021.103139 34111726

[9] Mulholland B, Goodall-Halliwell I, Wallace R, Chitiz L, Mckeown B, Rastan A, et al. Patterns of ongoing thought in the real world. Conscious Cogn. 2023;114:103530. doi: 10.1016/j.concog.2023.103530 37619452

[10] Smallwood J, Karapanagiotidis T, Ruby F, Medea B, de Caso I, Konishi M, et al. Representing Representation: Integration between the Temporal Lobe and the Posterior Cingulate Influences the Content and Form of Spontaneous Thought. PLoS One. 2016;11(4):e0152272. doi: 10.1371/journal.pone.0152272 27045292 PMC4821638

[11] Wallace RS, Mckeown B, Goodall-Halliwell I, Chitiz L, Forest P, Karapanagiotidis T, et al. Mapping patterns of thought onto brain activity during movie-watching. Elife. 2025;13:RP97731. doi: 10.7554/eLife.97731 39792001 PMC11723579

[12] Mckeown B, Strawson W, Zhang M, Turnbull A, Konu D, Karapanagiotidis T. Experience sampling reveals the role that covert goal states play in task-relevant behavior. Sci Rep. 2023;13(1):21710. 38066069 10.1038/s41598-023-48857-0 PMC10709616

[13] Mckeown B, Goodall-Halliwell I, Wallace R, Chitiz L, Mulholland B, Karapanagiotidis T, et al. Self-reports map the landscape of task states derived from brain imaging. Commun Psychol. 2025;3(1):8. doi: 10.1038/s44271-025-00184-y 39843761 PMC11754446

[14] Konu D, Turnbull A, Karapanagiotidis T, Wang H-T, Brown LR, Jefferies E, et al. A role for the ventromedial prefrontal cortex in self-generated episodic social cognition. Neuroimage. 2020;218:116977. doi: 10.1016/j.neuroimage.2020.116977 32450251 PMC7422831

[15] McVay JC, Kane MJ. Does mind wandering reflect executive function or executive failure? Comment on Smallwood and Schooler (2006) and Watkins (2008). 2010. 10.1037/a0018298 PMC2850105 20192557

[16] Chaieb L, Hoppe C, Fell J. Mind wandering and depression: A status report. Neurosci Biobehav Rev. 2022;133:104505. doi: 10.1016/j.neubiorev.2021.12.028 34929225

[17] Smallwood J, O’Connor RC. Imprisoned by the past: unhappy moods lead to a retrospective bias to mind wandering. Cogn Emot. 2011;25(8):1481–90. doi: 10.1080/02699931.2010.545263 21432633

[18] Forster S, Nunez Elizalde AO, Castle E, Bishop SJ. Unraveling the anxious mind: anxiety, worry, and frontal engagement in sustained attention versus off-task processing. Cereb Cortex. 2015;25(3):609–18. doi: 10.1093/cercor/bht248 24062316 PMC4318530

[19] Gaynor N, Fitzgerald L. Mind-wandering and its relationship with psychological wellbeing and obsessive-compulsive symptomatology in the context of covid-19. Psychol Rep. 2023. doi: 10.1177/00332941231203563 PMC12394777 37787173

[20] Rush AJ, Trivedi MH, Ibrahim HM, Carmody TJ, Arnow B, Klein DN, et al. The 16-Item Quick Inventory of Depressive Symptomatology (QIDS), clinician rating (QIDS-C), and self-report (QIDS-SR): a psychometric evaluation in patients with chronic major depression. Biol Psychiatry. 2003;54(5):573–83. doi: 10.1016/s0006-3223(02)01866-8 12946886

[21] Reiss S, Peterson RA, Gursky DM, McNally RJ. Anxiety sensitivity, anxiety frequency and the prediction of fearfulness. Behav Res Ther. 1986;24(1):1–8. doi: 10.1016/0005-7967(86)90143-9 3947307

[22] Baron-Cohen S, Wheelwright S, Skinner R, Martin J, Clubley E. The autism-spectrum quotient (AQ): evidence from asperger syndrome/high-functioning autism, males and females, scientists and mathematicians. J Autism Dev Disord. 2001;31:5–17. 11439754 10.1023/a:1005653411471

[23] Kessler RC, Adler L, Ames M, Demler O, Faraone S, Hiripi E, et al. The World Health Organization Adult ADHD Self-Report Scale (ASRS): a short screening scale for use in the general population. Psychol Med. 2005;35(2):245–56. doi: 10.1017/s0033291704002892 15841682

[24] Montgomery SA, Asberg M. A new depression scale designed to be sensitive to change. Br J Psychiatry. 1979;134:382–9. doi: 10.1192/bjp.134.4.382 444788

[25] Norman SB, Cissell SH, Means-Christensen AJ, Stein MB. Development and validation of an Overall Anxiety Severity And Impairment Scale (OASIS). Depress Anxiety. 2006;23(4):245–9. doi: 10.1002/da.20182 16688739

[26] Leon AC, Shear MK, Portera L, Klerman GL. Assessing impairment in patients with panic disorder: the Sheehan Disability Scale. Soc Psychiatry Psychiatr Epidemiol. 1992;27(2):78–82. doi: 10.1007/BF00788510 1594977

[27] Ware J, Sherbourne C. SHERBOURNE CD. THE MOS 36 ITEM SHORTFORM HEALTH SURVEY (SF-36). 1992. 1593914

[28] Development of the World Health Organization WHOQOL-BREF quality of life assessment. The WHOQOL Group. Psychol Med. 1998;28(3):551–8. doi: 10.1017/s0033291798006667 9626712

[29] Kahneman D, Krueger AB, Schkade DA, Schwarz N, Stone AA. A survey method for characterizing daily life experience: the day reconstruction method. Science. 2004;306(5702):1776–80. doi: 10.1126/science.1103572 15576620

[30] Mckeown B, Poerio GL, Strawson WH, Martinon LM, Riby LM, Jefferies E, et al. The impact of social isolation and changes in work patterns on ongoing thought during the first COVID-19 lockdown in the United Kingdom. Proc Natl Acad Sci USA. 2021;118(40):e2102565118. doi: 10.1073/pnas.2102565118 34599096 PMC8501798

[31] Poerio GL, Smallwood J. Daydreaming to navigate the social world: what we know, what we don’t know, and why it matters. Soc Pers Psychol Compass. 2016;10(11):605–18.

[32] Fell J, Chaieb L, Hoppe C. Mind wandering in anxiety disorders: A status report. Neurosci Biobehav Rev. 2023;155:105432. doi: 10.1016/j.neubiorev.2023.105432 37898447

[33] Smallwood J, Schooler J. The science of mind wandering: empirically navigating the stream of consciousness. Annu Rev Psychol. 2015;66(1):487–518. 25293689 10.1146/annurev-psych-010814-015331

[34] Klinger E, Cox W. Handbook of motivational counseling: concepts, approaches, and assessment. John Wiley & Sons. 2003.

[35] Chitiz L, Mckeown B, Mulholland B, Wallace R, Goodall-Halliwell I, Ho N. Mapping cognition across lab and daily life using experience-sampling. 2023. 10.1016/j.concog.2025.103853 40209288

